# Multiplex Detection of Homo- and Heterodimerization of G Protein-Coupled Receptors by Proximity Biotinylation

**DOI:** 10.1371/journal.pone.0093646

**Published:** 2014-04-01

**Authors:** Elisabeth Steel, Victoria L. Murray, Allen P. Liu

**Affiliations:** 1 Department of Mechanical Engineering, University of Michigan, Ann Arbor, Michigan, United States of America; 2 Department of Biomedical Engineering, University of Michigan, Ann Arbor, Michigan, United States of America; 3 Cellular and Molecular Biology Program, University of Michigan, Ann Arbor, Michigan, United States of America; 4 Biophysics Program, University of Michigan, Ann Arbor, Michigan, United States of America; Medical School of Hannover, Germany

## Abstract

Dimerization of G protein-coupled receptors (GPCRs) represents a potential mechanism by which GPCR functions are regulated. Several resonance energy transfer (RET)-based methods have revealed GPCR homo- and heterodimerization. However, interpretation of an increase in FRET efficiency could be attributed to either dimerization/oligomerization events or conformational changes within an already dimerized/oligomerized receptor complex. Furthermore, RET-based methods can only measure pairwise dimerization, and cannot easily achieve multiplex detection. In this study, we applied proximity-based biotinylation for detecting receptor dimerization by utilizing a specific enzyme-substrate pair that are fused to GPCRs. The biotin ligase BirA is fused to CXCR4 and site-specifically biotinylates an acceptor peptide (AP) in the presence of biotin. As a test case for our newly developed assay, we have characterized the homo-dimerization of chemokine receptor CXCR4 and heterodimerization of CXCR4 with CCR2 or CCR5. The degree of biotinylation varies with the amount of GPCR-AP as well as biotinylation time. Using enzyme/substrate receptor pairs and measuring receptor biotinylation, we demonstrate that CXCR4 can homo-dimerize and hetero-dimerize with CCR2 and CCR5. The effect of CXCL12, agonist for CXCR4, was found to decrease surface biotinylation of CXCR4-AP. This effect is due to a combination of CXCR4 endocytosis and stabilization of CXCR4 homodimers. Finally, when CXCR4-AP, CCR2-AP, and CCR5-AP were expressed together, we observed CXCR4-CXCR4 homodimers and CXCR4-CCR2 and CXCR4-CCR5 heterodimers. The newly developed assay opens new opportunity for multiplex detection for GPCR homo- and heterodimerization within the same cellular context.

## Introduction

GPCRs mediate the majority of our physiological responses to neurotransmitters, hormones, and environmental stimulants by their capacity to engage in diverse signaling pathways [1]. Dimerization/oligomerization between GPCRs is recognized to modulate the pharmacological characteristics of the receptors and influence their coupling to G proteins [2]. Plasma membrane receptors can interact with each other forming either homo- or heterodimers, depending on the identity of the interacting receptors. Recent studies have shown that GPCRs can exist as dimers or as part of larger oligomeric complexes [3], [4]; however the functional significance of dimerization remains poorly understood. Furthermore, there is increasing evidence that homo- and heterodimerization of activated GPCRs represents a means to control the specificity and increase the diversity of signaling events [5]. While Class B GPCR are obligate dimers [6], most class A GPCRs are capable of functioning as single units or form homo- or heterodimers [7], [8].

Co-immunoprecipitation is arguably the most utilized biochemical technique for determining protein-protein interactions, and one of the few techniques that does not require expression of exogenous protein products. Although the technique is still commonly used, it requires solubilization of membrane proteins, thereby generally providing little information on the sub-cellular localization of protein-protein interaction. These assays also generally cannot distinguish between direct or indirect interactions, and the equilibrium condition for immunoprecipitation will identify the most abundant interactions, leaving out transiently interacting binding proteins. Several sophisticated techniques have been developed in recent years to complement traditional biochemical techniques. Both fluorescence resonance energy transfer (FRET) and bioluminescence resonance energy transfer (BRET) technology have been widely used to authenticate the proximity of proteins in living cells [9], [10]. The approach is typically based on the use of fusion proteins with resonance energy transfer-compatible GFP variants as an acceptor while the energy donor can be a fluorescent protein or bioluminescence from an enzyme [11]. The efficiency of energy transfer is highly dependent on the distance between the energy donor and the energy acceptor and varies inversely with *R^6^* where *R* is the distance between the donor and acceptor. Because both FRET and BRET are proximity-based methods, an increase in energy transfer efficiency (i.e. the ratio of emissions between donor and acceptor) can be associated with receptor dimerization/oligomerization. A limitation typically associated with resonance energy transfer techniques is the small dynamic range [12]. Another limitation for the use of resonance energy transfer techniques stems from more recent application of FRET in reporting conformational changes in GPCRs. Different FRET efficiencies reported in these experiments were interpreted as different receptor conformations [13]. Relative affinity can be discerned from RET50 determination, providing insight into protomer propensity to interact with one another, whereas maximal RET signal reports conformational changes [8]. However, when considering an increase in FRET efficiency, it is difficult to discern between dimerization/oligomerization events from a change of conformation within already dimerized/oligomerized receptor complexes.

Presented by the challenges of interpreting FRET or BRET data, we sought out to develop a technique based on proximity biotinylation for detecting receptor homo- and heterodimerization. Our method could uniquely measure dimerization on the plasma membrane and also enables multiplex detection of homo- and heterodimerization within the same cellular context. In this study, we have applied our assay to investigate chemokine receptor homo- and heterodimerization centering on CXCR4, CCR2, and CCR5.

## Materials and Methods

### Cell culture and constructs

Recombinant adenoviruses encoding human transferrin receptor (TfnR), beta 2 adrenergic receptor (β2AR), CXCR4, CCR2, or CCR5 were generated by placing them using overlap PCR into a tetracycline transactivator (tTA)-regulated adenoviral vector. The acceptor peptide (AP) was fused at the N terminus to each of the chemokine receptors and β2AR, and at the C terminus of the TfnR. A separate CXCR4 expressing adenovirus was generated with the biotin ligase BirA fused to the N terminus. All constructs containing AP also has an HA tag preceding it, and the BirA-CXCR4 construct has a FLAG tag preceding the BirA.

Retinal pigment epithelial wild type cells (RPE WT, ATCC CRL-2302, gift from Dr. Sandra Schmid, UTSW) were maintained in F-12/DMEM (Gibco) and supplemented with 10% fetal bovine serum (Sigma), 100 units/ml penicillin and streptomycin, and 20 mM HEPES. When seeded for experiments, cells were cultured in DMEM supplemented with 10% charcoal/dextran treated fetal bovine serum (Thermo Scientific), 100 units/ml penicillin and streptomycin, and 20 mM HEPES. Cells were co-infected for 18 hours with tTA adenovirus and adenovirus containing a tetracycline-regulatable promoter (Tet-off) and encoding a protein of interest. In this current study, no tetracycline was added.

### Flow cytometry

RPE WT were cultured and infected as described above. After 18 hr infection, cells were incubated with a biotinylation media consisting of DMEM, 100 μM biotin, 1 mM ATP, and 5 mM MgCl_2_ for a designated time period (i.e. 5, 10, 15, or 30 minutes) at 37 degrees. The cells were then rinsed with PBS, detached using citric saline buffer, pelleted and resuspended in 1% BSA/PBS solution. The remaining steps were all performed on ice. After blocking in 1% BSA/PBS solution for 15 minutes, the cells were dual labeled in 1% BSA/PBS solution with a primary anti-HA antibody (Roche) for 30 minutes followed by secondary anti-mouse Alexa Fluor 647 and streptavidin-phycoerythrin (PE) (Invitrogen) for 30 minutes. Cells were rinsed using PBS and fixed in 4% paraformaldehyde. Following fixation, cells were rinsed with PBS, pelleted, and resuspended in 0.5% BSA/PBS. Permeabilized samples were first fixed in 4% paraformaldehyde then incubated with PBS containing 0.1% Triton X-100 for 15 minutes at room temperature before proceeding with the labeling steps as previously described.

Flow cytometry was performed on a Beckman-Coulter Cyan or an Invitrogen Attune and accompanying Summit software or Attune Cytometer Software. PE was excited by the 488 nm laser and the emission was collected using the 575/24 filter. The Alexa Fluor 647 was excited by the 635 nm laser and the emission was collected using the 665/20 filter. Forward scatter, side scatter, and emission were collected for a minimum of 10,000 cells for each sample. Gating on the forward versus side scatter plot eliminated debris and doublet cells. Positive fluorescence was determined by gating compared to a negative control. The percent positive and median were multiplied to calculate signal intensity.

### On-cell Western

RPE WT were seeded in two rows of a 96-well plate at a density of 20,000 cells/well; one row was designated for labeling of surface receptors, the other row for labeling total receptors after permeabilization. After 18 h of adenoviral infection, the cells were rinsed with PBS and fixed with 4% paraformaldehyde. All wells were washed three times with PBS. Additionally, the sample wells to be permeabilized were washed four times with PBS containing 0.1% Triton X-100 for 5 minutes with moderate shaking. Cells in all wells were incubated in a 1% BSA/PBS blocking solution at room temperature for 15 minutes. All cells were incubated with primary mouse anti-HA antibody (1∶5000, Roche) in 1% BSA/PBS for 1 hour at room temperature. The 1% BSA/PBS labeling solution for the permeabilized sample wells contained 0.1% Tween 20. After washing the plate three times with PBS, all cells were incubated with a secondary labeling solution containing goat anti-mouse IRDye-800CW antibody (Li-Cor) and TO-PRO-3 (Invitrogen) nuclear stain for 1 hour at room temperature. Triplicate samples receiving no labeling solution serve as background subtraction. Following this, the plate was washed five times with PBS containing 0.1% Tween 20 for 5 minutes at room temperature with gentle shaking and then three more times with PBS. Wash solution was completely removed by inversion then the plate was centrifuged at 300 g for 15 seconds. The plate was scanned using a Li-Cor Odyssey Sa Infrared Imager at 200 μm resolution, 3 mm offset, and intensity 7 for channels 700 and 800. Analysis was performed using Li-Cor Image Studio software. Anti-HA IRDye-800 signal was calculated after cell number normalization using the TO-PRO-3 signal collected in channel 700.

### Immunofluorescence imaging

RPE WT were cultured on coverslips, infected with adenoviruses for 18 hours and incubated in biotinylation media for 15 minutes at 37 degrees. Coverslips were washed with PBS and fixed in 4% paraformaldehyde for 20 minutes. Cells were labeled with streptavidin conjugated Alexa Fluor 568 (Invitrogen) for 1 hour. Fixed samples were imaged on a fluorescence microscope (model Eclipse Ti; Nikon) using a 60x objective with a sCMOS camera (model C11446 Orca Flash 4.0; Hamamatsu) and equipped with motorized excitation and emission filter wheels (Sutter Instrument Co.). Image acquisition was performed using Micro-Manager. Image processing was performed using ImageJ.

### Western Blot

RPE WT were cultured in a 6-well plate, infected with adenoviruses for 18 hr, and incubated in biotinylation media for 15 minutes at 37 degrees. Cells were washed with PBS and lysis buffer was applied for 10 minutes on ice. A cell scraper was used to dislodge cells and the lysates were transferred to microtubes on ice. After cell debris was spun down using a microcentrifuge, the lysate supernatants were combined with SDS loading buffer, treated with 1 mM DTT for 10 minutes at room temperature, and then loaded in duplicate into a 10% SDS-PAGE gel for electrophoresis at 90 V for 2 hours. The protein bands were transferred to a nitrocellulose membrane and blocked with Odyssey Blocking Buffer for 1 hour (Li-Cor). One set of samples was probed for protein expression with primary mouse anti-HA antibody (1∶5000, Roche) and rabbit anti-actin (1∶3000, Pierce) followed by secondary labeling solution containing goat anti-mouse IRDye-800CW antibody and anti-rabbit IRDye-680RD (Li-Cor). The second set of samples was probed for biotinylation with Streptavidin Alexa Fluor 750 (1∶10,000). The membranes were scanned using a Li-Cor Odyssey Sa Infrared Imager at 200 μm resolution, 3 mm offset, and intensity 7 for channels 700 and 800. For Figure 4C, band intensity analysis was quantified using Li-Cor Image Studio software. Streptavidin (SA) signal was calculated such that the normalized SA signal was equal to the western blot SA band intensity divided by the surface receptor percentage found by the on cell western multiplied by the western blot HA band intensity.

### Statistics

Analysis of variance (ANOVA) tests for fixed factors were performed followed by Tukey multiple comparison procedures to detect differences between group streptavidin signal means. When homogeneity of variance was not met, a Kruskal Wallis test was performed according to the method described by Zar followed by a nonparametric Tukey-type multiple comparison procedure. There were equal numbers of samples in each group. Statistical significance was considered for α = 0.05. For the Kruskal Wallis and Tukey multiple comparison tests, H_c_ and *q* values greater than the critical value for the appropriate degrees of freedom and α = 0.05 are considered significant.

## Results

### Characterization of proximity biotinylation assay for chemokine receptor dimerization

**Figure 1:**
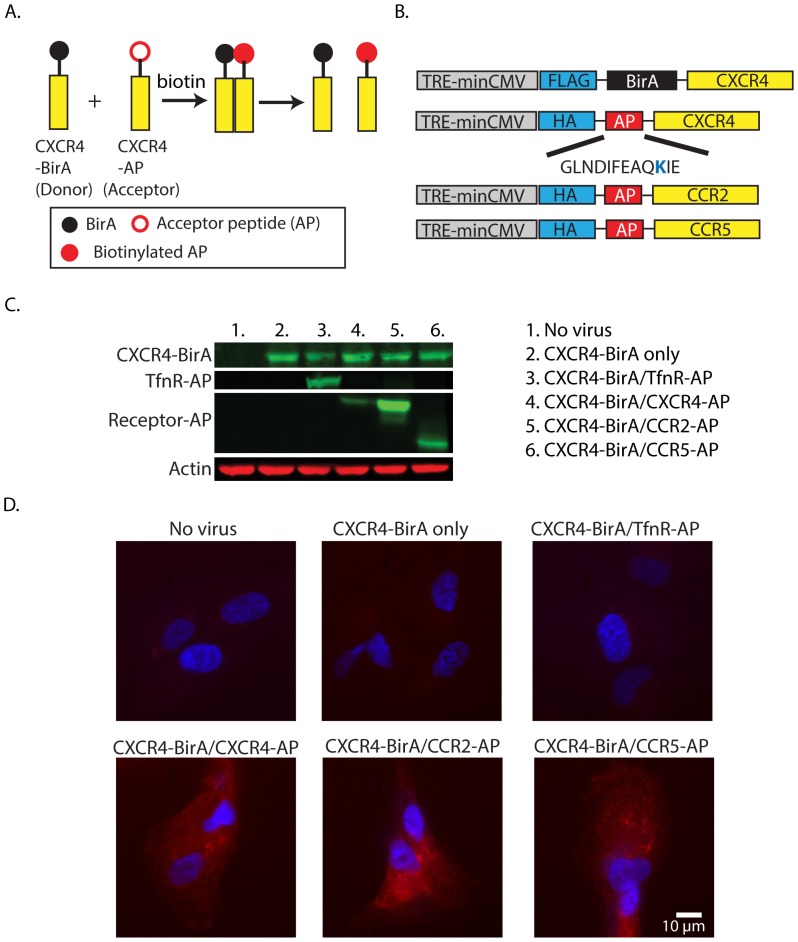
Proximity biotinylation for detecting receptor dimerization. A) Schematic of biotinylation of CXCR4-AP by CXCR4-BirA. CXCR4-BirA is akin of donor and CXCR4-AP is akin of acceptor as in FRET assays. B) Schematic of adenovirus constructs of CXCR4-BirA, CXCR4-AP, CCR2-AP, and CCR5-AP. C) Western blot of CXCR4 from RPE cells infected with different adenoviruses used in this study. CXCR4-BirA was probed with anti-CXCR4 antibodies, and the AP receptors were probed with anti-HA antibodies. D) Fluorescence images of AlexaFluor 568 SA in RPE cells expressing CXCR4-BirA and receptor-AP and treated with biotin for 15 minutes. Samples with no virus, CXCR4-BirA, CXCR4-BirA/TfnR-AP all have background staining while samples with CXCR4-BirA and either of CXCR4-AP, CCR2-AP, or CCR5-AP all exhibited significant fluorescent labeling.

To demonstrate the concept of using proximity biotinylation for receptor dimerization, several receptors fusions were created in an adenovirus vector (Figure 1). The use of adenovirus vectors offers the advantage that infection efficiency is high. For biotinylation, we have adopted the use of BirA, a biotin ligase from *E. coli* that recognizes and biotinylates the lysine residue on a specific acceptor peptide (AP) sequence (GLNDIFEAQKIE) [14], [15]. The BirA and AP were fused to the extracellular domains of our receptors with short flexible linkers (GSGSTSGSGK) inserted to ensure high probability of enzyme-substrate interaction (Figure 1A and B).

To measure receptor dimerization, it is important to use cells that express low endogenous levels of receptors of interests. Using flow cytometry and a cell line that expresses CXCR4 or CCR5 as positive controls, we found that retinal pigment epithelial (RPE) cells contain undetectable levels of CXCR4, CCR2, and CCR5 and are deemed suitable for the current study (Figure S1). The fetal bovine serum (FBS) that is used for routine tissue culture contains greater than 20 nM of biotin, according to the manufacturer's specification. Although this is a low level of biotin, the presence of 10% FBS during the virus infection steps to express the receptor constructs was sufficient to biotinylate the receptors without exogenously added biotin. To circumvent this problem, charcoal/dextran treated FBS that contains less than 2 nM of biotin was used, and this yielded no detectable biotinylation without addition of exogenous biotin (Figure S2). Charcoal/dextran treated FBS was used in all experiments unless otherwise noted.

To check the surface expression of our chemokine receptor constructs, we developed an on-cell western (OCW) protocol for 96 well plate to be used with an infrared imaging system. Cells were fixed and labeled with anti-HA either with or without permeabilization to determine total and surfaced expressed receptors, respectively. Fluorescence signals were scanned and normalized to a nuclear staining dye to account to cell density variability between wells. Between 35–40% of the chemokine receptors were expressed on the cell surface (Figure S3). To measure receptor dimerization, biotin and ATP were added to cells infected with pairs of CXCR4-BirA and receptor-AP. Cells expressing CXCR4-BirA and receptor-AP were verified by Western blot (Figure 1C). To detect biotinylation, Alexa Fluor 568 labeled streptavidin (AF568 SA) was used to label biotinylated receptors (Figure 1D). In cases where no virus was added or only CXCR4-BirA virus was used, cells did not have specific staining. As a negative control, we have used transferrin receptor-AP (TfnR-AP) that is not expected to dimerize with CXCR4. Indeed, TfnR was not biotinylated in cells expressing CXCR4-BirA/TfnR-AP. For CXCR4-AP, CCR2-AP, and CCR5-AP following biotinylation, immunofluorescence experiments showed that these constructs were biotinylated. In addition, we also examined possible heterodimerization between CXCR4 and a non-chemokine GPCR, β2 adrenergic receptor (β2AR). Although not widely studied, activated CXCR4 has been shown to physically interact with β2AR and regulate its downstream signaling pathway in rat cardiac myocytes [16]. This interaction has been shown previously using co-immunoprecipitation, confocal microscopy, and BRET and we have confirmed this interaction using our proximity biotinylation assay (Figure S4).

### Quantitative analysis of receptor dimerization by proximity biotinylation

**Figure 2:**
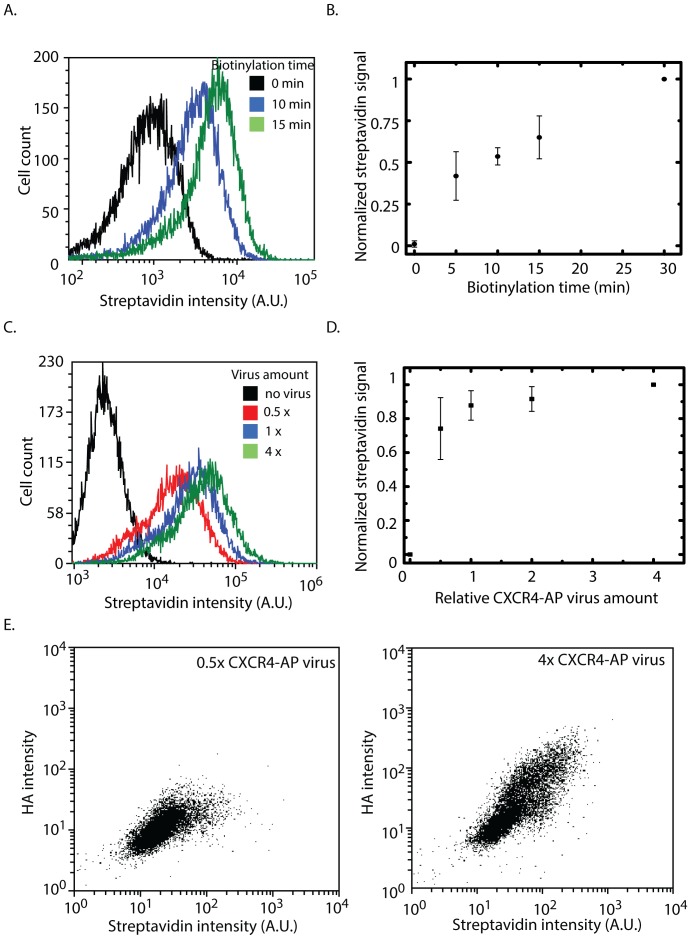
Characterization of proximity biotinylation of CXCR4 homodimerization. A) Intensity histogram of CXCR4 biotinylation with increasing biotinylation time for a representative experiment. B) Fluorescence signal increases with the amount of time cells are incubated with biotin before fixation and labeling. Intensity are computed as % positive x median intensity of % positive and normalized by value at 30 min (n = 5, mean ± S.E.). C) Intensity histogram of CXCR4 biotinylation with increasing virus amount for a representative experiment. D) Percent positive cells labeled with SA-PE increases with relative CXCR4-AP adenovirus load measured by flow cytometry after 15 minute of biotinylation. (n = 3, mean ± S.E., p = 0.094) E) Scatter plots of anti-HA and SA-PE in cells expressing CXCR4-BirA and CXCR4-AP. The GPCR-AP constructs have HA epitope tags to facilitate measurement of GPCR-AP expression. There is a positive correlation between CXCR4 biotinylation and CXCR4-AP expression within a presumably heterogeneous cell population.

Next, different parameters that could affect the biotinylation were systematically explored. First, the amount of biotinylation should vary with the amount of biotinylation time of the expressed receptor-AP. Using flow cytometry to detect surface labeled biotinylation with streptavidin-phycoerythrin (SA-PE), we observed the degree of labeling depends on biotinylation time since biotinylated receptors would remain biotinylated even in the case that the dimerized receptors would dissociate. Indeed, increasing biotinylation time increased the amount of biotinylated CXCR4 (Figure 2A and 2B). It is unlikely that this increase in biotinylation is due to the catalysis time for AP biotinylation since k_cat_ is ∼0.5 min^−1^ [15]. Instead, this is likely to reflect the continuous association and dissociation of receptors in monomer-dimer dynamic equilibrium [17]. It is interesting that the biotinylation increased linearly with time and does not seem to reach saturation after 30 minutes, and this will be further discussed later. Akin to changing the acceptor/donor ratio in BRET or FRET applications, we used different amount of adenoviruses to alter expression level. We observed increased labeling of biotinylated CXCR4 with increasing dosage of CXCR4-AP adenoviruses (Figure 2C and 2D). While there was an increase in the degree of biotinylation as CXCR4-AP was increased, the dynamic range was quite small (8 fold increase in virus amount resulted in ∼50% increase in biotinylation). From our result that biotinylation increased with time and that we held our biotinylation to 15 minutes, the degree of labeling was limited by time, such that under the same BirA expression, increasing CXCR4-AP had a smaller effect. Although small, the degree of biotinylation depended on the expression of GPCR-AP and since there was a variable expression among a cell population, we performed dual label flow cytometry experiment using SA-PE and surface immunolabeling of HA-tagged CXCR4-AP. As we expected and confirming earlier results, cells expressing more CXCR4-AP were more biotinylated across different amount of virus added (Figure 2E).

### CXCR4 homodimerization and CXCR4 heterodimerization

**Figure 3:**
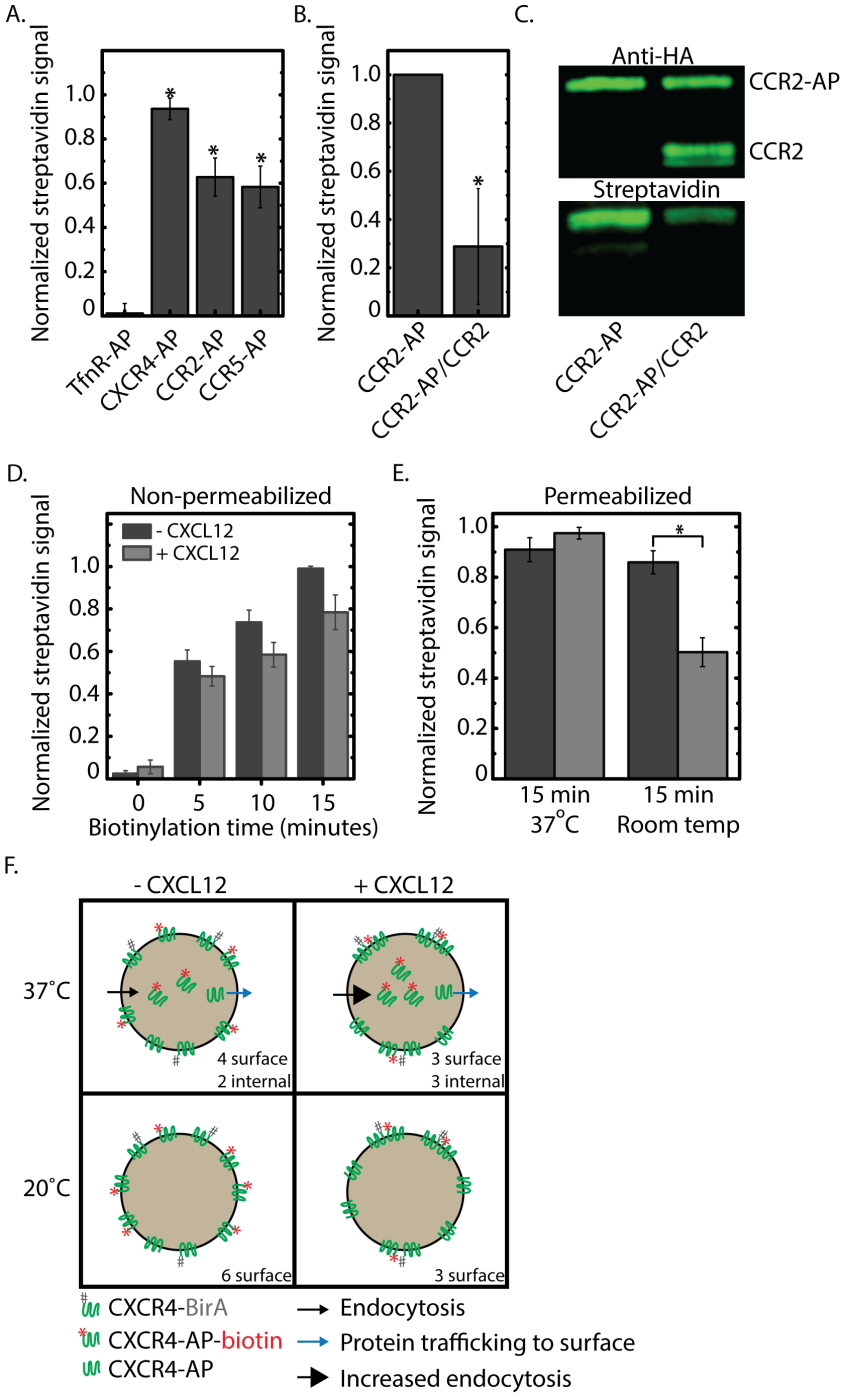
CXCR4 homodimerization vs. CXCR4 heterodimerization. A) Measurement of biotinylation of different chemokine receptor pairs with a flow cytometer. Signal represents the product of percent positive cells and median intensity of positive cells. CXCR4 homodimers and CXCR4/CCR2 and CXCR4/CCR5 heterodimers were observed after 5 minutes biotinylation. Negative controls included no virus and BirA virus only. No virus condition was used to gate for the positive cells and BirA virus only had ∼2% positive, which is subtracted off as background signal for all the samples. (n = 8, mean ± S.E.) When homogeneity of variance was not met, a Kruskal Wallis test was performed according to the method described by Zar [6], in which a significant effect of receptor pair on biotinylation signal was found (Hc = 19.84) considered for the critical value X^2^(2) = 14.067 (p<0.05, ν = 7). A nonparametric Tukey-type multiple comparison test showed significant differences (critical value q(1) = 2.218, p<0.05) between all groups: TfnR-AP vs. CXCR4-AP (q = 51.5), TfnR-AP vs. CCR2-AP (q = 34.1), TfnR-AP vs. CCR5-AP (q = 30.2), CXCR4-AP vs. CCR2-AP (q = 17.4), CXCR4-AP vs. CCR5-AP (q = 21.3), CCR2-AP vs. CCR5-AP (q = 3.9.) A q value greater than the critical value is significant. B) Expression of CCR2 decreased the biotinylation of CCR2-AP in a flow cytometry experiment (p = 0.049). C) Western blot of biotinylation of CCR2-AP in the presence and absence of competing CCR2-no AP. D) Effect of CXCL12 on CXCR4 homodimerization. CXCL12 tends to reduce the biotinylation of CXCR4-AP at 37 deg for all time points in non-permeabilized cells (2 Factor ANOVA (Time x CXCL12 Treatment), p<0.001 for Time, p = 0.015 for CXCL12 Treatment, Interaction p = 0.182). Signals are calculated as in A). (n = 6, mean ± S.E.) E) Cells were biotinylated at 37 degrees or at room temperature for 15 minutes. Cells were permeabilized and biotinylated receptors were measured by labeled streptavidin (n = 5, mean ± S.E.). Signals are calculated as in A). 2 Factor ANOVA (Temperature x CXCL12 Treatment) revealed a significant interaction (p<0.001). Significant differences revealed by Tukey multiple comparison tests are denoted by an asterisk (p<0.05). F) Schematic explaining the effect of CXCL12 on homodimerization at 37 degrees and room temperature. At 37°C, the addition of CXCL12 increases CXCR4 internalization while unlabeled receptors continue to traffic to the surface. At room temperature, both endocytosis and new receptor delivery are decreased. The addition of CXCL12 stabilizes CXCR4 dimers, reducing the availability of CXCR4-BirA to dimerize and biotinylate other CXCR4-AP receptors available on the surface.

To evaluate the degree of CXCR4 homodimerization and CXCR4 heterodimerization with CCR2 and CCR5, dual channel flow cytometry measurements were performed for cells expressing pairs of CXCR4-BirA and a GPCR-AP. SA-PE signals were normalized by anti-HA signals to properly account varying GPCR-AP expression between different conditions. Furthermore, we used the product of percent positive and median signal of the positive-gated cells as a metric for comparison. The rational is that percent positive may not reveal the full detail of biotinylation as the intensity distribution of the positive-gated cells is missing from such commonly reported measurements. Similar to the immunofluorescence data, expression with a control receptor TfnR both showed little biotinylation. By comparison, expression of CXCR4-AP, CCR2-AP, or CCR5-AP together with CXCR4-BirA, all showed substantial degree of biotinylation (Figure 3A). Interestingly, our assay revealed CXCR4-CCR5 heterodimers, where there has been inconsistent literature [18]–[20]. To ascertain that the biotinylation of GPCR-AP represents *bona fide* receptor heterodimerization, we generated CCR2 construct lacking fused AP. We showed that CCR2-no AP could effectively compete with CCR2-AP, leading to a marked decrease in CCR2 biotinylation (Figure 3B). This is further confirmed by Western blot analysis showing the reduction in CCR2 biotinylation in cells expressing both CCR2-AP and CCR2-no AP compared to cells expressing CCR2-AP (Figure 3C). Together, these experiments demonstrated the specificity of the biotinylation towards only AP fused receptors.

To investigate whether CXCR4 agonist C-X-C motif ligand 12 (CXCL12) would affect homodimerization of CXCR4, biotinylation experiments were performed in the presence and absence of 5 nM CXCL12 for different amount of biotinylation time (Figure 3D). We found that surface biotinylated CXCR4 was reduced by the addition of CXCL12 at all the time points. Since CXCL12 is known to induce CXCR4 internalization through clathrin-mediated endocytosis [21], the reduction in surface biotinylation could be in part due to the internalization of biotinylated receptors. Indeed, CXCL12-treated cells had comparable level of total biotinylated receptors measured with permeabilized samples at 37 degrees (Figure 3E), indicating the effect of CXCL12 induced CXCR4 endocytosis. Since endocytosis is heavily temperature dependent [22], we carried out the biotinylation experiment at room temperature (∼20°C) which would significantly reduce endocytosis. Interestingly, in the presence of CXCL12, there was a significant decrease in the biotinylation (p<0.001), suggesting that there might be a second contributor to the reduction in surface biotinylation of CXCR4-AP. We attribute this decrease to the stabilization of CXCR4 dimers which increased dimer lifetime leading to less rounds of biotinylation. These results are summarized in a schematic that illustrates how CXCL12 reduced surface biotinylated CXCR4 at 37 degrees and room temperature (Figure 3F). More specifically, in a permeabilized sample at 37 degrees, there would be little difference in the detected biotinylated CXCR4. However, in a non-permeabilized sample where only surface receptors were measured, increased endocytosis in CXCL12-treated cells would lead to a reduction in biotinylated CXCR4. At room temperature, where membrane trafficking slowed down significantly, the reduction in biotinylation in the presence of CXCL12 supported the idea that CXCR4 dimers were stabilized by CXCL12.

### Multiplex assay for receptor dimerization

**Figure 4:**
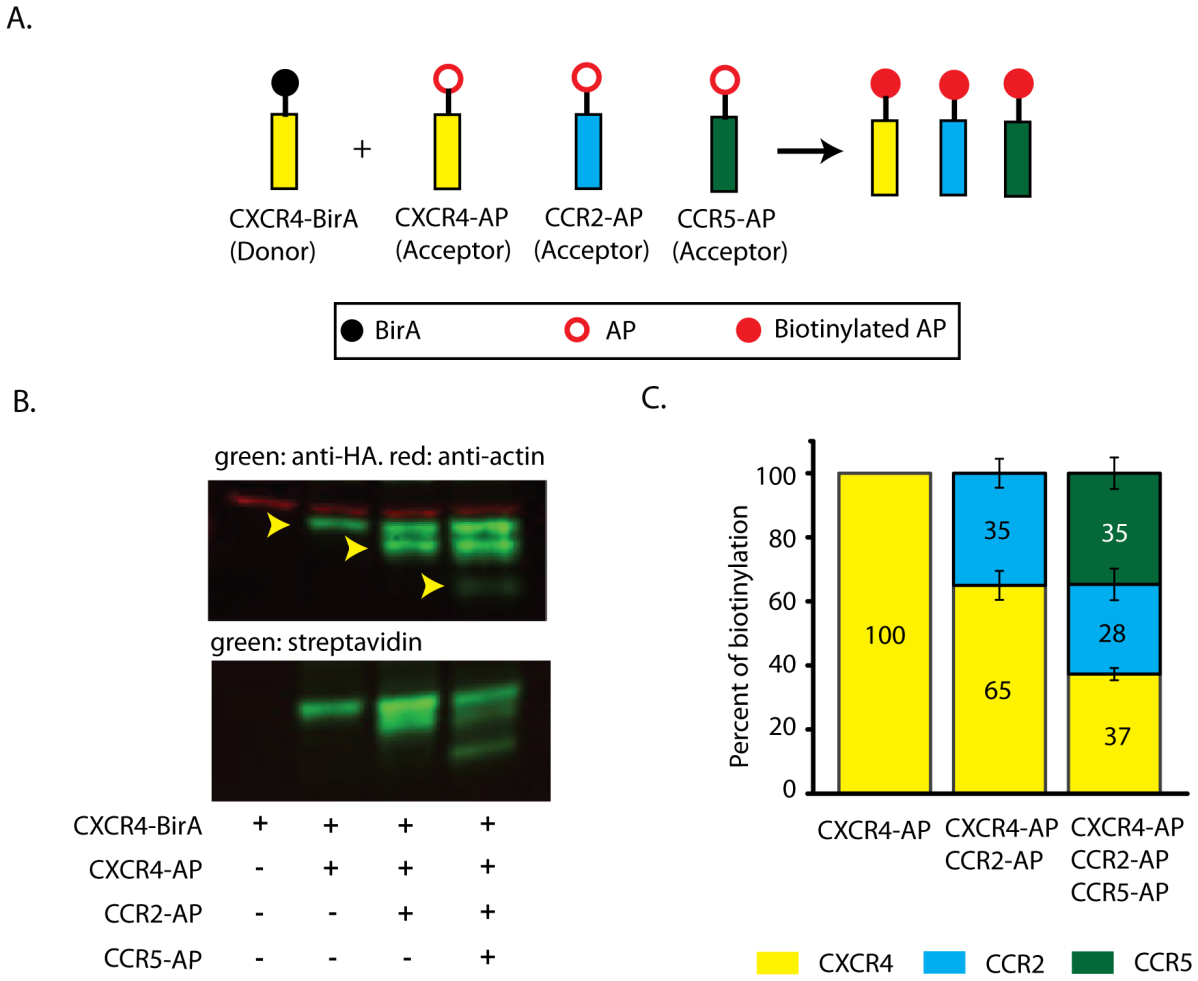
Multiplex biotinylation by CXCR4-BirA for measuring homo- and heterodimerization within the same cellular context. A) Schematic of the multiplex assay of CXCR4-BirA and CXCR4-AP, CCR2-AP, and CCR5-AP. B) Fluorescence Western blot of different combination of GPCR-AP helps delineate the specific band for each chemokine receptor. C) Analysis of the proportion relative to total biotinylation for each combination of GPCR-AP in B). Intensity of the bands was normalized to surface expressed receptors obtained from another independent experiment. (n = 4, mean ± S.E.) An arcsine transformation was performed in order to run a one factor ANOVA on the relative percentages obtained from quantifying western blot band intensities (amount of CXCR4 homodimer greater than CCR2-CXCR4 heterodimer, p = 0.001; no difference between dimer proportions containing CXCR4-AP, CCR2-AP, CCR5-AP, p = 0.087).

One of the most notable advantages of our biotinylation assay is the ability for multiplex detection for receptor dimerization. Analogous to donor and acceptor in FRET, BirA and AP can be viewed as donor and acceptor, respectively. In FRET, it would be very difficult to resolve FRET signals using a single fluorescence donor and multiple acceptors, though bimolecular fluorescence complementation FRET can circumvent this shortcoming [23]. In our scheme, a single CXCR4-BirA can biotinylate multiple receptors that are fused to AP, in this case, CXCR4-AP, CCR2-AP, and CCR5-AP (Figure 4A). Subsequently, separation and identification of the biotinylated receptors could reveal the differential homodimerization and heterodimerization of CXCR4. Although CXCR4, CCR2, and CCR5 have similar molecular weights (39,745, 41,914 and 40,524 Da respectively), the three biotinylated receptors could be resolved on a fluorescent Western blot (Figure 4B). We were able to deduce the bands by systematically removing one receptor-AP and look for the difference in band patterns. CXCR4-AP, CCR2-AP, and CCR5-AP had similar levels of surface expression relative to the total from our OCW experiments (Figure S3) and this information was used to determine the surface receptor expression for the Western blot data. In RPE cells that expressed all three receptor-AP constructs, we detected the highest proportion of biotinylated CXCR4-CXCR4 followed by CXCR4-CCR5 then CXCR4-CCR2 dimerization but no significant difference between pairs (p = 0.087). When only CXCR4-AP and CCR2-AP were expressed along with CXCR4-BirA, we found more CXCR4 homodimer compared CXCR4-CCR2 heterodimer (p = 0.001) (Figure 4C).

## Discussion

We have previously used the BirA/AP biotinylation system to site-specifically labeled TfnR for live cell imaging [24]. Others have used this system as well for imaging receptor oligomerization during endocytosis and for detecting transcellular protein-protein interactions [25], [26]. The specificity of this enzyme substrate system prompted us to investigate the possibility of using this for detecting protein-protein interaction, and specifically for examining homo- and heterodimerization of GPCRs. The premise of this assay is that only dimers of a certain lifetime would yield biotinylation since there is a probability that the receptor-AP can be biotinylated when it encounters the receptor-BirA. We do not think random collision would account for significant amount of the measured signals as TfnR-AP was not significantly biotinylated in any of our assays. Although there are size differences between the extracellular domains of CXCR4 and TfnR, a structural analysis suggested that TfnR-AP could be biotinylated by CXCR4-BirA (Figure S5; PDB ID: 1CX8 [27], PDB ID: 1BIA [28]). The continuous increase of biotinylation over a long period of time would suggest that our assay does not reach saturation. We do not believe this is due to non-specific binding because the amount of time for streptavidin labeling is fixed. While this may seem like a disadvantage as an assay, we think this could be an intrinsic characteristic of our assay in that the efficiency of biotinylation is low and that we are capturing a percentage of dimerized receptors. Prolonged biotinylation time is perhaps a drawback as new receptors may continue to be made during the time though endocytosis is also expected to internalize biotinylated receptors. A way to increase biotinylation efficiency may be to increase ATP concentration. Increasing biotin concentration, however, would increase non-specific binding [14]. The complete labeling of all receptor-AP would simply report the total receptors on the cell surface, while short term labeling was sufficient for us to detect receptor dimerization.

In this work, we focus on three chemokine receptors, CXCR4, CCR2, and CCR5. There are conflicting data in the literature regarding the existence of either homo- or heterodimers and whether these interactions are constitutive or ligand-induced. Vila-Coro *et al*. showed that CXCL12 triggered CXCR4 dimerization whereas Babcock *et al*. found that CXCR4 exists as constitutive dimers [18], [29]. Using protein fragment complementation based on firefly luciferase, it was shown that CXCR4 molecules constitutively dimerize [30]. Despite a ∼70% sequence identity and close structural homology between CCR2 and CCR5, it is not entirely clear whether this predisposes equal propensity for CXCR4 to heterodimerize with either receptor. CXCR4 has been reported to form heterodimer with CCR2 [10], but not with CCR5 [18], [19]. There is also indication that CXCR4 does not dimerize with wild type CCR2, but with a mutant CCR2 [31], as well as literature pointing to heterodimerization of CXCR4 with CCR5 [32], [33]. High resolution electron microscopy seems to support the finding that CXCR4 and CCR5 do not heterodimerize as the receptors were detected in clusters that are spatially segregated [34]. However, these studies have been performed in different cell lines and employing different techniques, so comparison is not straightforward. By using a cell line that does not express endogenous receptors under study, our assumption is that receptor dimerization represents physical interactions between two receptors that it does not require specific cellular adaptor proteins to mediate this interaction. Finally, most of the established assays do not differentiate between cell surface and intracellular receptor dimerization. Using our biotinylation assay for detecting receptor dimerization on cell surface, we found that CXCR4 homodimerizes in the absence of CXCL12, consistent with several previous studies [18], [30]. However, there was a reduction in surface biotinylated CXCR4 in the presence of CXCL12 at 37 degrees in non-permeabilized cells, but not in permeabilized cells, which suggests CXCR4 is internalized in CXCL12-treated cells. At room temperature, when vesicle trafficking (including endocytosis) is significantly reduced, we observed a reduction in CXCR4 biotinylation with CXCL12 addition. From this, we concluded that CXCL12 promotes CXCR4 internalization, a view that is consistent with current understanding of CXCR4 function [35], as well as stabilizes homodimers of CXCR4.

Comparison of ligand-based and sequence-based dendrograms, it was recently found that CXCR4, CCR1, CCR2, and CCR5 belong to a group based on ligand similarity [36]. This lends strong support that within the same cellular context, there could be a complex composition of homodimers and heterodimers. However, few studies have attempted to address this. Multiplexed FRET has been demonstrated using quantum dots in configuration of either multiple donors or multiple acceptors. However, it is difficult to conceive how this could be applied to cellular measurements as deconvolution of time-resolved spectra is required for data analysis [37]. Using a combination of luminescence complementation and BRET, it was found that there exist hetero-oligomeric complexes composed of at least three chemokine receptors CCR2, CCR4, and CXCR4 [38]. Our multiplex assay measures the degree of biotinylation between different receptor pairs but cannot distinguish oligomeric complexes. From our results, we determined that CXCR4 homodimers, CXCR4-CCR5, and CXCR4-CCR2 heterodimers form. In the context of previous finding and ours is that there are different proportions of homo- and heterodimers, though we cannot exclude the possibility of coexisting oligomeric and dimeric species. Nonetheless, our assay provides a new means to measure receptor dimerization among multiple species within the same cell and can be broadly applied to other receptor systems.

Elucidation and manipulation of chemokine receptor homodimers and heterodimers may offer new therapeutic possibilities [39]. Bivalent CXCR4 ligand that binds to CXCR4 with higher affinity has been synthesized and it has been shown to have enhanced antiviral activity [40]. For a heterodimer, it is perhaps possible to activate one of the two monomeric units to trigger internalization of both receptors and/or initiate signaling of the G proteins that it binds to. Conversely, a defective monomer may adversely affect the function of the dimer, as the primarily heterozygous disease WHIM syndrome (warts, hypogammaglobulinemia, infections, and myelokathexis syndrome) presents with coexpression of a truncated CXCR4 that exerts dominance over the expressed wild-type receptor [41]. Thus, receptor dimerization may be a mechanism to achieve signal diversity. Such crosstalk between different receptors is expected to add another level of specificity for the fine-tuning of cellular responses.

## Supporting Information

Figure S1 **RPE cells have no endogenous CXCR4, CCR2, and CCR5.** Positive controls using NP-2 cells stably expressing CD4/CXCR4 (A) or CD4/CCR5 (B) showed that our flow cytometry assay works. On the other hand, RPE cells (C) do not appear to have detectable level of CXCR4, CCR2, or CCR5 by flow cytometry.
(TIF)

Figure S2 **Comparison of regular FBS and charcoal dextran-treated FBS in biotinylation in the absence of exogenous biotin.** Biotinylation is clearly present when cells are cultured in regular FBS (top panels), whereas no labeling is observed when cells are cultured in charcoal dextran-treated FBS. This demonstrates the low level of biotin in regular-FBS containing media could biotinylate receptor pairs during the virus infection step.
(TIF)

Figure S3 **Surface expression of CXCR4-AP, CCR2-AP, and CCR5-AP determined by On Cell Western (OCW).** A) OCW image of cells in 96 well plate expressing the indicated viruses stained with anti-HA antibody or a nuclear-stained dye (for normalization of cell density). Anti-HA antibody was either added to label cell surface receptor (top row) or in permeabilized cells to label total receptor (bottom row). B) Quantification of surface to total receptor for CXCR4-AP, CCR2-AP, and CCR5-AP (n = 3, mean ± S.D.)
(TIF)

Figure S4 **Heterodimerization between CXCR4 and β2AR.** A) Western blot of β2AR from RPE cells infected with CXCR4-BirA and β2AR-AP adenoviruses. One blot was probed for anti-HA to visualize expression of β2AR, a separate blot was probed with Streptavidin-750 which indicated β2AR biotinylation. B) Fluorescence images of AlexaFluor 568 SA in RPE cells expressing CXCR4-BirA and β2AR-AP and treated with biotin for 15 minutes. β2AR-AP exhibited fluorescent labeling compared to the no virus staining. C) Intensity histogram of β2AR biotinylation using flow cytometry.
(TIF)

Figure S5 **Structural analysis of CXCR4-BirA and TfnR-AP. Both the N-terminus of CXCR4 (39 amino acids) and the last twelve amino acids of TfnR C-terminus do not have any defined secondary structure allowing for flexibility of movement.** A schematic of the crystal structure of BirA (PDB ID: 1BIA) in close proximity to the crystal structure of TfnR (PDB ID: 1CX8, one chain) is shown here The 10 aa peptide linker off the N-terminus end of CXCR4 was modeled with a helix secondary structure. The same process was repeated for the AP linker attached to TfnR. We used PyMol to measure distances from end to end while in helix or beta-sheet formation. Two internal measurements were made within BirA and TfnR to be used as reference. The distance from the N-terminus of BirA to the residues involved in biotinylating AP sequences is 22 Å. The distance from the N-terminus of the TfnR to the last helical residue nearest the C-terminus is 31.3 Å. Although we cannot know for certain the final structure of the AP linker attached to TfnR, but it seems feasible that TfnR-AP could be biotinylated if they were in fact to dimerize.
(TIF)
