# Supplementary figures and images for: Multiplex Detection of Homo- and Heterodimerization of G Protein-Coupled Receptors by Proximity Biotinylation
Source: PLoS One. 2014 Apr 1;9(4):e93646. doi: 10.1371/journal.pone.0093646 (PMC3972117; doi:10.1371/journal.pone.0093646)

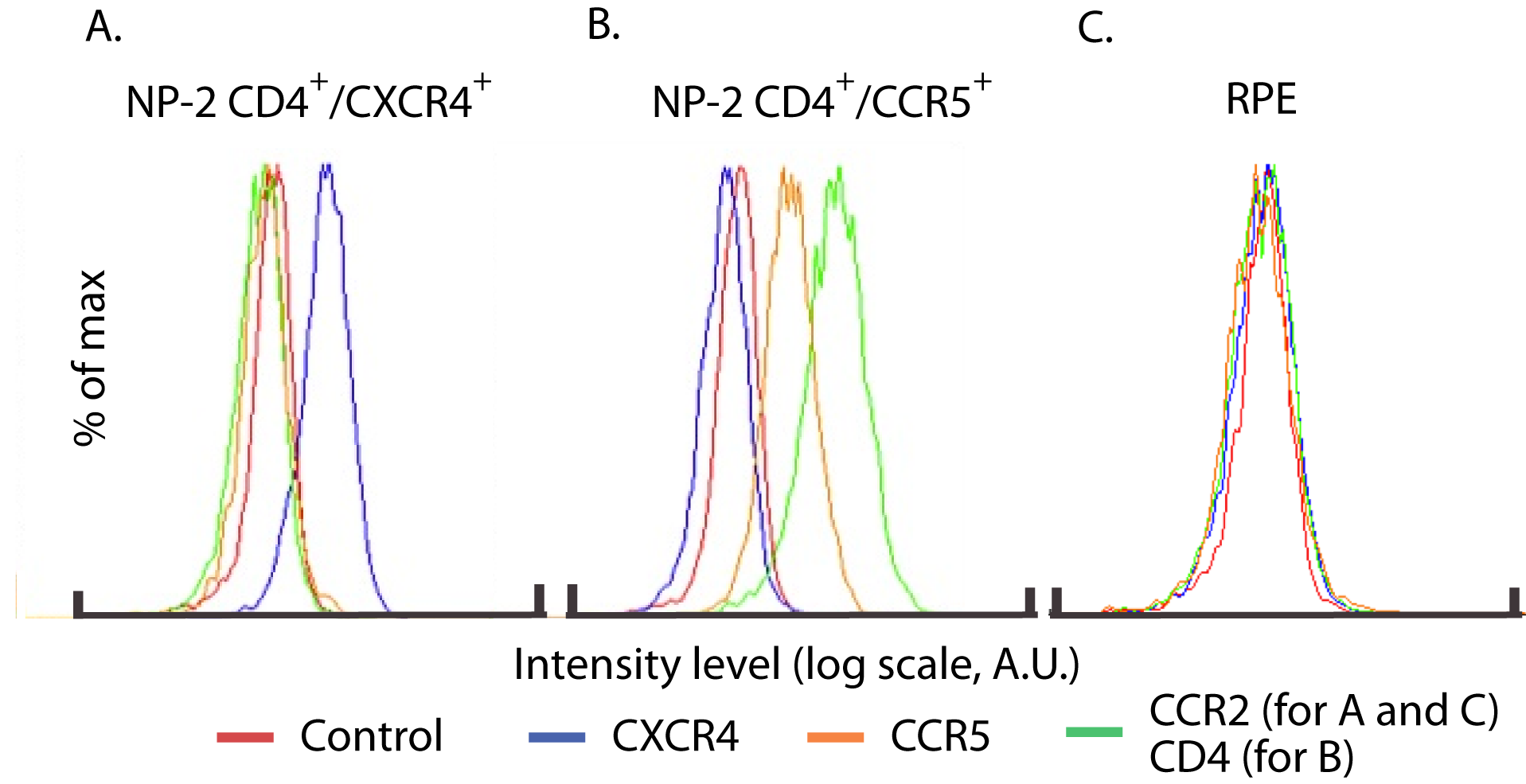

Supplement: Figure S1 — RPE cells have no endogenous CXCR4, CCR2, and CCR5. Positive controls using NP-2 cells stably expressing CD4/CXCR4 (A) or CD4/CCR5 (B) showed that our flow cytometry assay works. On the other hand, RPE cells (C) do not appear to have detectable level of CXCR4, CCR2, or CCR5 by flow cytometry. (TIF) [file pone.0093646.s001.tif]

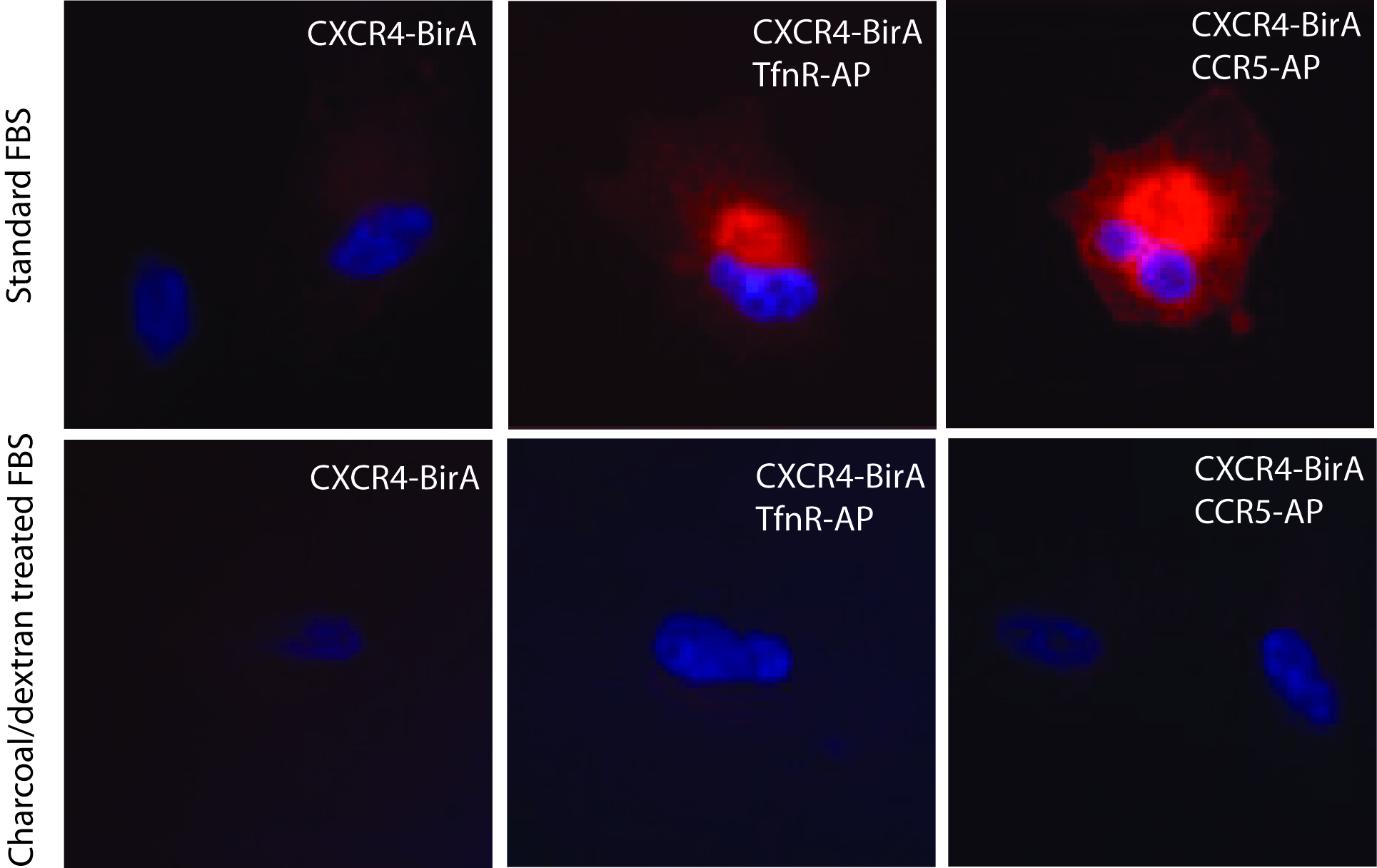

Supplement: Figure S2 — Comparison of regular FBS and charcoal dextran-treated FBS in biotinylation in the absence of exogenous biotin. Biotinylation is clearly present when cells are cultured in regular FBS (top panels), whereas no labeling is observed when cells are cultured in charcoal dextran-treated FBS. This demonstrates the low level of biotin in regular-FBS containing media could biotinylate receptor pairs during the virus infection step. (TIF) [file pone.0093646.s002.tif]

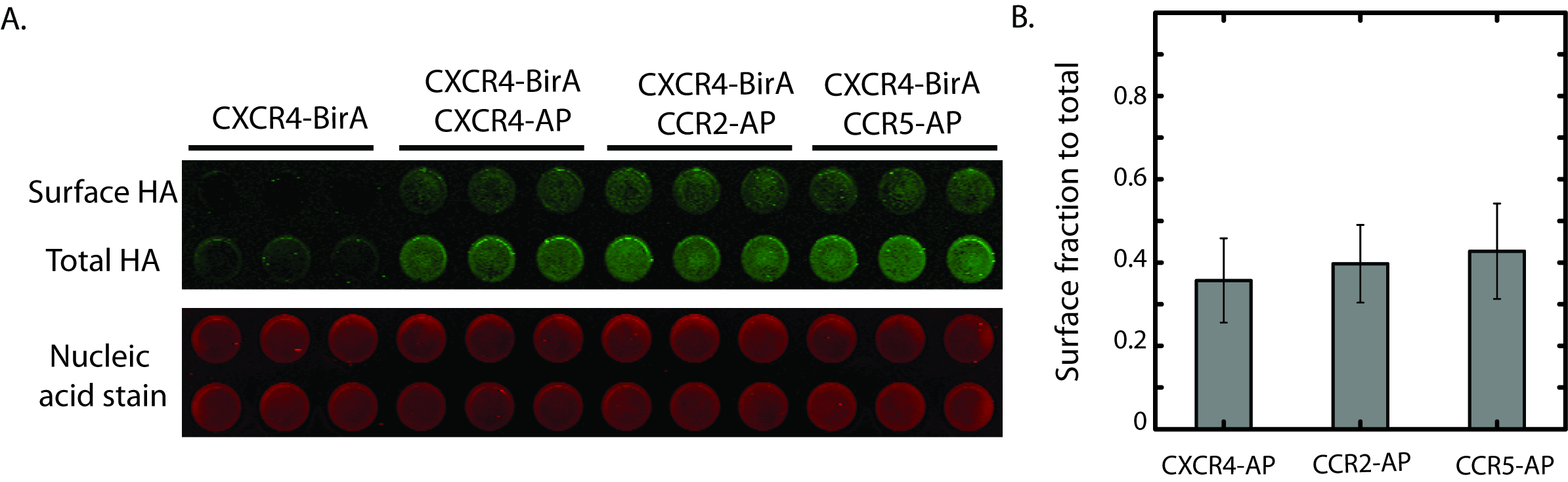

Supplement: Figure S3 — Surface expression of CXCR4-AP, CCR2-AP, and CCR5-AP determined by On Cell Western (OCW). A) OCW image of cells in 96 well plate expressing the indicated viruses stained with anti-HA antibody or a nuclear-stained dye (for normalization of cell density). Anti-HA antibody was either added to label cell surface receptor (top row) or in permeabilized cells to label total receptor (bottom row). B) Quantification of surface to total receptor for CXCR4-AP, CCR2-AP, and CCR5-AP (n = 3, mean ± S.D.) (TIF) [file pone.0093646.s003.tif]

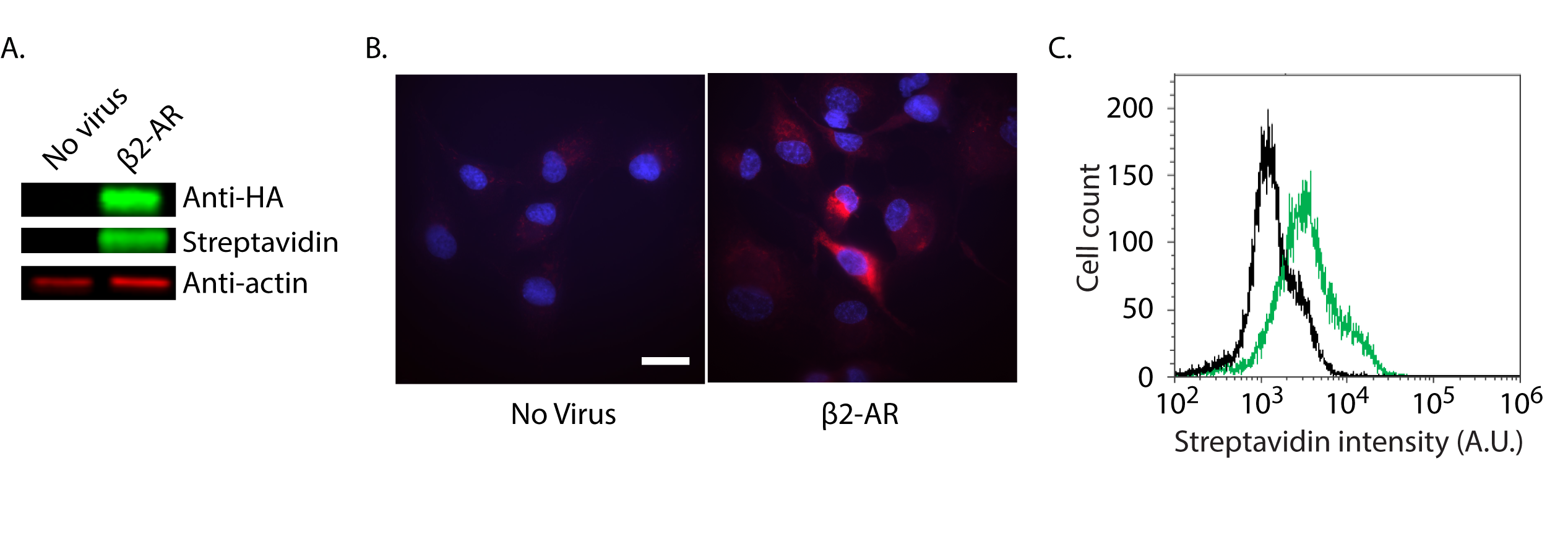

Supplement: Figure S4 — Heterodimerization between CXCR4 and β2AR. A) Western blot of β2AR from RPE cells infected with CXCR4-BirA and β2AR-AP adenoviruses. One blot was probed for anti-HA to visualize expression of β2AR, a separate blot was probed with Streptavidin-750 which indicated β2AR biotinylation. B) Fluorescence images of AlexaFluor 568 SA in RPE cells expressing CXCR4-BirA and β2AR-AP and treated with biotin for 15 minutes. β2AR-AP exhibited fluorescent labeling compared to the no virus staining. C) Intensity histogram of β2AR biotinylation using flow cytometry. (TIF) [file pone.0093646.s004.tif]

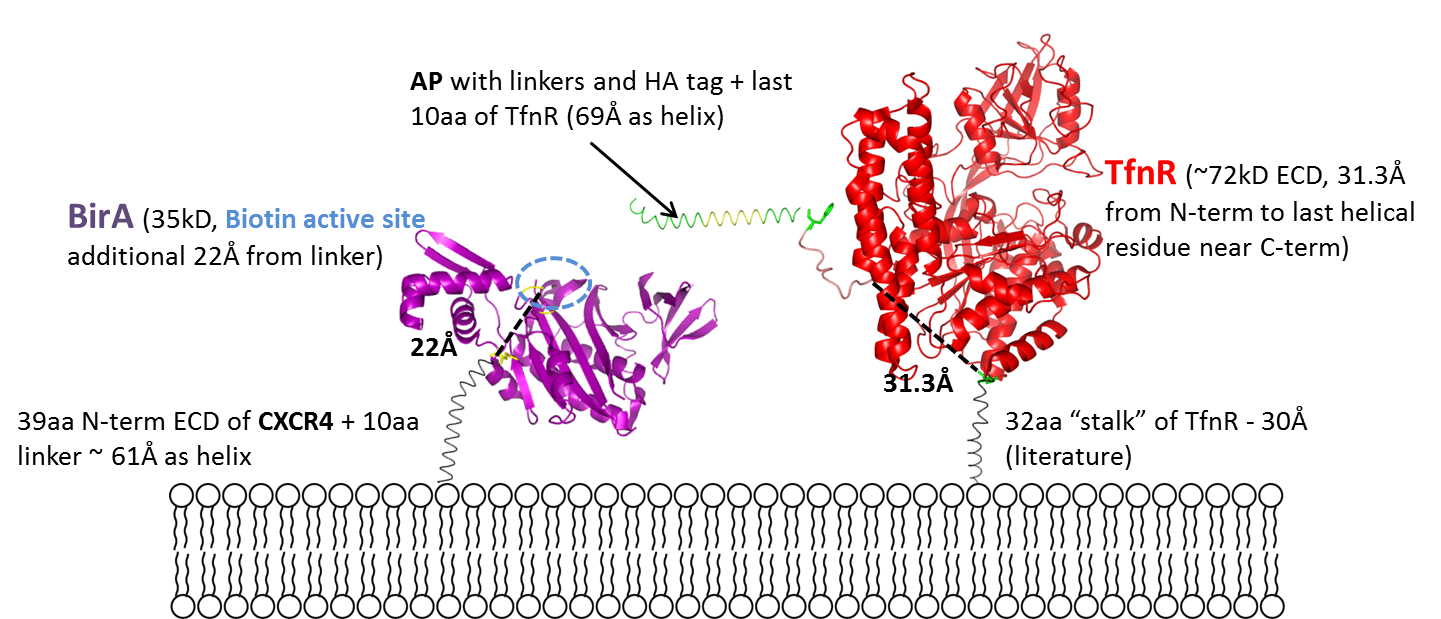

Supplement: Figure S5 — Structural analysis of CXCR4-BirA and TfnR-AP. Both the N-terminus of CXCR4 (39 amino acids) and the last twelve amino acids of TfnR C-terminus do not have any defined secondary structure allowing for flexibility of movement. A schematic of the crystal structure of BirA (PDB ID: 1BIA) in close proximity to the crystal structure of TfnR (PDB ID: 1CX8, one chain) is shown here The 10 aa peptide linker off the N-terminus end of CXCR4 was modeled with a helix secondary structure. The same process was repeated for the AP linker attached to TfnR. We used PyMol to measure distances from end to end while in helix or beta-sheet formation. Two internal measurements were made within BirA and TfnR to be used as reference. The distance from the N-terminus of BirA to the residues involved in biotinylating AP sequences is 22 Å. The distance from the N-terminus of the TfnR to the last helical residue nearest the C-terminus is 31.3 Å. Although we cannot know for certain the final structure of the AP linker attached to TfnR, but it seems feasible that TfnR-AP could be biotinylated if they were in fact to dimerize. (TIF) [file pone.0093646.s005.tif]
